# Ferroptosis-associated signaling pathways and therapeutic approaches in depression

**DOI:** 10.3389/fnins.2025.1559597

**Published:** 2025-03-19

**Authors:** Xuyang Feng, Wenyu Zhang, Xiaoxi Liu, Qiuxuan Wang, Xiao Dang, Jingxian Han, Xuezhu Zhang

**Affiliations:** ^1^First Teaching Hospital of Tianjin University of Traditional Chinese Medicine, Tianjin, China; ^2^National Clinical Research Center for Chinese Medicine Acupuncture and Moxibustion, Tianjin, China; ^3^Tianjin University of Traditional Chinese Medicine, Tianjin, China

**Keywords:** depression, ferroptosis, signaling pathways, traditional Chinese medicine, acupuncture

## Abstract

Ferroptosis, a newly identified form of cell death, is characterized by excessive iron accumulation and lipid peroxidation. Studies indicate a strong association between ferroptosis and depression; however, the precise signaling pathways and underlying molecular mechanisms remain unclear. This review summarizes the role of ferroptosis in depression and its associated signaling pathways. Additionally, therapeutic approaches for depression based on ferroptosis theory are reviewed, providing novel targets for the prevention and treatment of depression and laying a foundation for future research on the relationship between ferroptosis and depression.

## 1 Introduction

**Figure 1:**
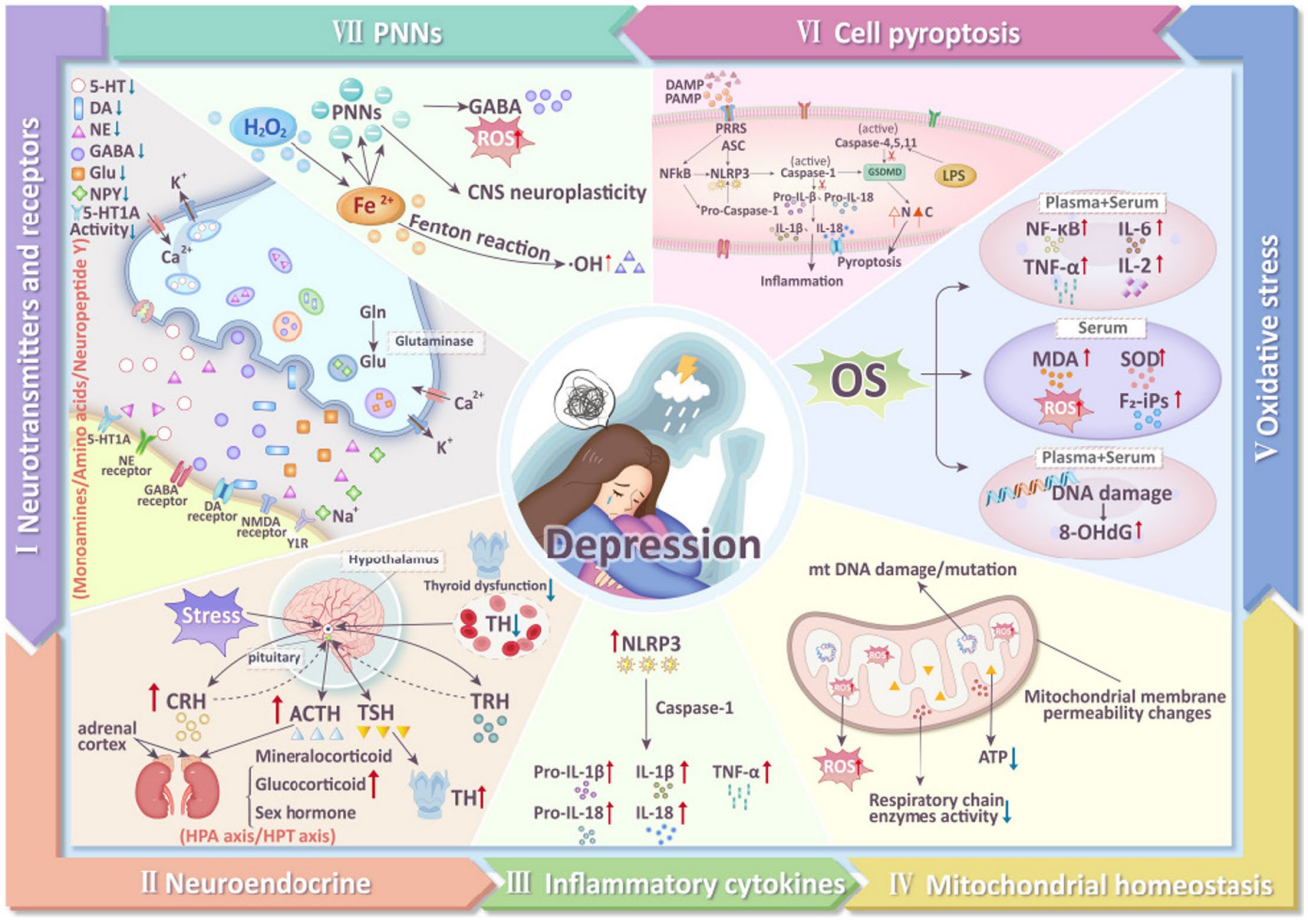
Mechanisms of depression (5-HT, 5-hydroxytryptamine; DA, dopamine; NE, norepinephrine; GABA, γ-aminobutyric acid; Glu, Glutamate; Gln, Glutamine; NMDA, N-methyl-D-aspartate receptor; TH, thyroid hormone; TRH, thyrotropin-releasing hormone; TSH, thyroid stimulating hormone; ACTH, adrenocorticotropic hormone; CRH, corticotropin releasing hormone; NLRP3, NLR Family, Pyrin Domain Containing 3; Pro-IL- 1β, Pro-Interleukin 1β; Pro-IL-18, Pro-Interleukin 18; IL-18, interleukin-18; IL-1β, interleukin-1β; TNF-α, tumor necrosis factor-α; ROS, reactive oxygen species; 8-OHdG, 8-hydroxydeoxyguanosine; MDA, malondialdehyde; SOD, superoxide dismutase; F2-iPs, F2-isoprostanes; IL-2, interleukin-2; IL-6, interleukin-6; GSDMD, gasdermin D; LPS, Lipopolysaccharide; PNNs, perineuronal nets).

Depression is a major global health issue, characterized by pervasive, chronic, and recurrent episodes of depressed mood and agitation (Du et al., 2023), often accompanied by symptoms such as negative attitudes, reduced appetite, and pain (Bair et al., 2003). In recent years, the prevalence of depression has been rising, with incidence in men approximately half that in women, while the suicide rate in men is 3–4 times higher than in women (Swetlitz, 2021). Depression is also a leading cause of disability worldwide. In 2022, the World Health Organization (WHO) reported a 25% increase in global cases of anxiety and depression following the COVID-19 pandemic, affecting approximately 300 million people worldwide (Lucero-Prisno et al., 2023; Zhou et al., 2021). By 2030, depression is projected to be one of the primary causes of mortality globally (Tian et al., 2013). Currently, two major challenges remain in addressing depression. First, there is no definitive cure; international approaches primarily involve pharmacotherapy and psychological counseling, which are often associated with delayed effectiveness and significant side effects. Second, the pathogenesis of depression is still not clearly understood. Multiple factors are believed to contribute to the development of depression, including neurotransmitter metabolism (Schildkraut et al., 1972), neuroendocrine function (Min et al., 2012), inflammatory cytokines (Raison et al., 2006), mitochondrial function (Song et al., 2023), oxidative stress (Bhatt et al., 2020), pyroptosis (Li et al., 2021), and the perineuronal net (PNN) (Yu et al., 2020) (Figure 1). Therefore, exploring the pathophysiological mechanisms of depression and identifying effective treatments are urgent priorities.

**Figure 2:**
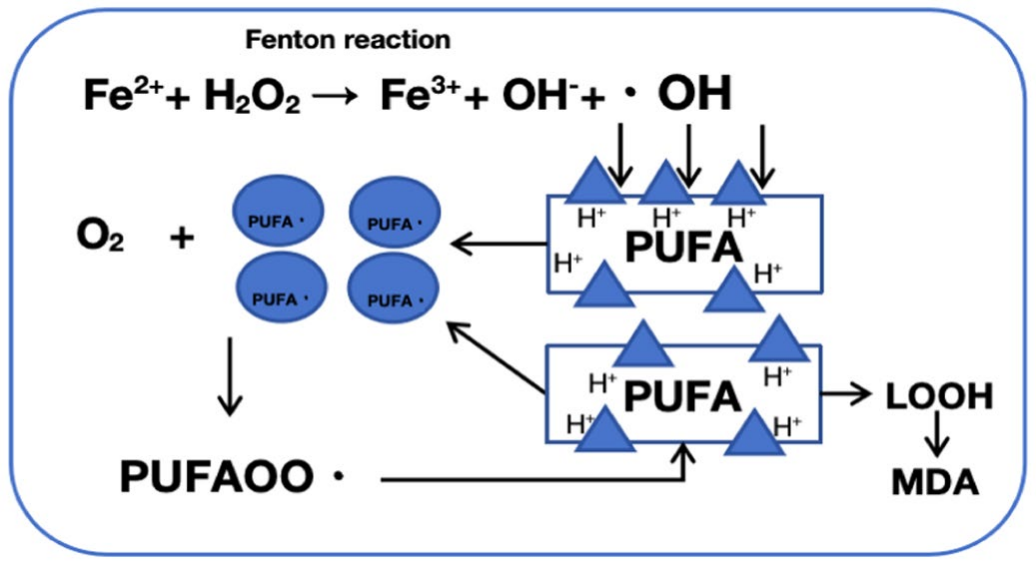
Mechanism of fenton reaction-induced phospholipid peroxidation: a schematic representation. The Fenton reaction involves the catalysis of hydrogen peroxide (H_2_O_2_) decomposition by ferrous ions (Fe^2+^), generating highly reactive hydroxyl radicals (·OH) and hydroxide ions (OH^−^). These hydroxyl radicals are potent oxidants that attack lipid molecules within cells, abstracting a hydrogen atom and forming lipid radicals (PUFA·). Lipid radicals react with oxygen to produce peroxyl radicals (PUFAOO·), which subsequently attack neighboring lipid molecules, initiating a chain reaction of lipid peroxidation, leading to the formation of lipid hydroperoxides (LOOH). Lipid hydroperoxides (LOOH) are unstable and can decompose, generating more free radicals or breaking down into aldehydes (such as malondialdehyde, MDA), ultimately causing cellular damage and dysfunction.

**Figure 3:**
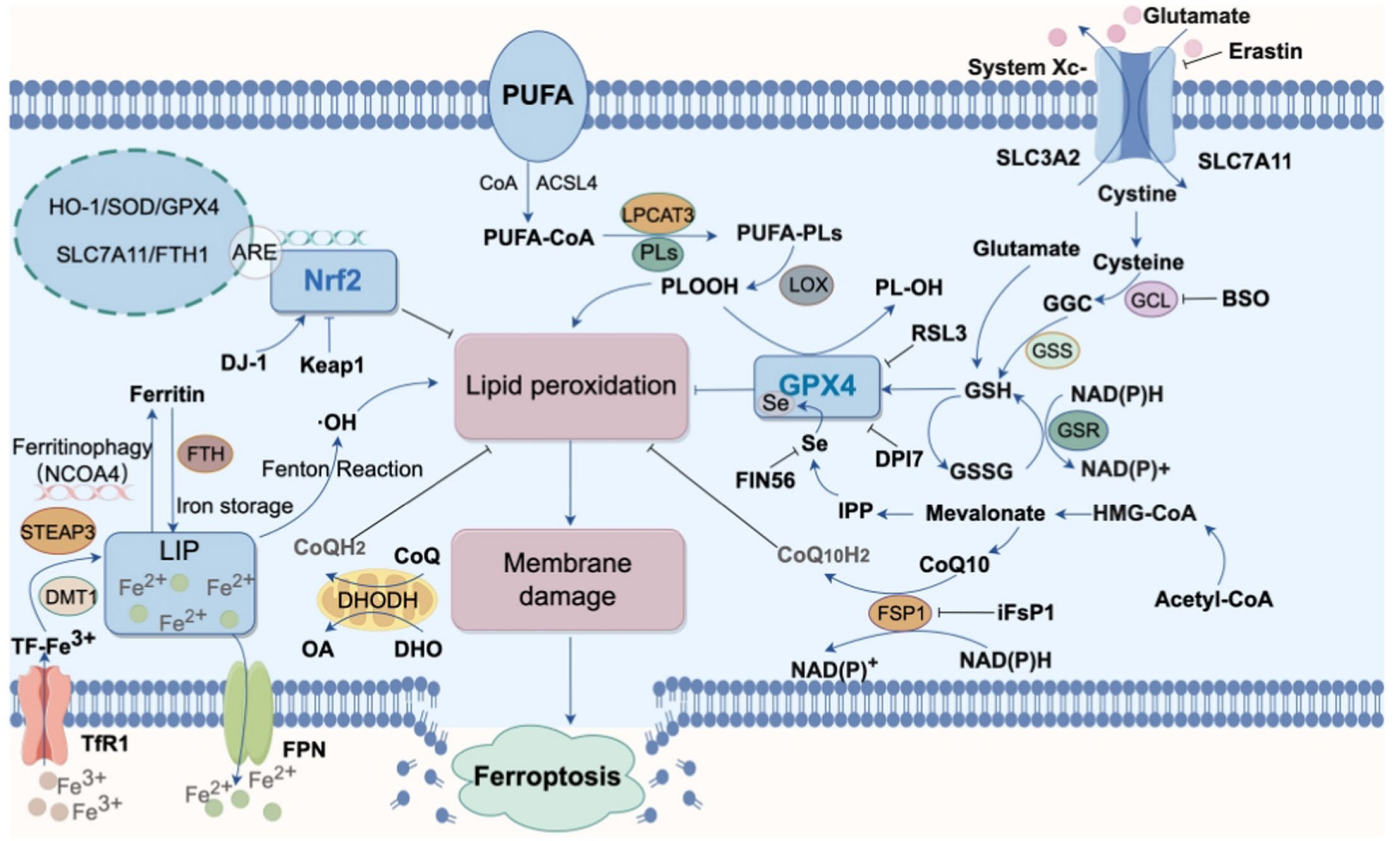
Main pathways of ferroptosis. Various pathways promote intracellular lipid peroxidation, leading to cell membrane rupture and subsequent ferroptosis. The processes associated with the occurrence of ferroptosis include cysteine metabolism, iron metabolism, inhibition of GPX4, suppression of NRF2 activity, and oxidation of polyunsaturated fatty acids (PUFAs) (STEAP3, six transmembrane epithelial antigen of the prostate3; LIP, labile iron pool; DMT1, divalent metal ion transporter 1; FTH, Ferritin Heavy chain; TfR1, Transferrin receptor 1; ACSL4, acyl-CoA synthetase long-chain family member 4; CoQ10, coenzyme Q10; DHODH, dihydroorotate dehydrogenase; GPX4, glutathione peroxidase 4; GSH, glutathione; GCL, glutamate-cysteine ligase;GGC, γ-glutamylcysteine; GSSG, glutathione (Oxidized); GSR, glutathione reductase; GSS, glutathione synthetase CoQ10H2, ubiquinol; DHO, Dihydroorotate; FSP1, ferroptosis suppressor protein 1HO-1, heme oxygenase-1; LPCAT3, lysophosphatidylcholine acyltransferase 3; Nrf2, nuclear factor erythroid 2-related factor 2; PL-OOH, phospholipid hydroperoxide; PUFA, polyunsaturated fatty acid; PUFA-PL, phospholipid containing polyunsaturated fatty acid chain; ROS, reactive oxygen species; BSO, buthionine sulfoximine; IPP, isopentenyl pyrophosphate;Keap1, kelch-like ECH-associated protein 1; FPN, Ferroportin; NCOA4, the nuclear receptor coactivator 4).

In 2012, Dixon et al. (2012) first proposed the concept of “ferroptosis,” an iron-dependent form of cell death. Ferroptosis is defined by excessive intracellular iron accumulation due to metabolic imbalance, where Fe^2+^ generates a large amount of hydroxyl radicals (OH) through the Fenton reaction (Figure 2). This process catalyzes the oxidation of polyunsaturated fatty acids (PUFAs) in cell membranes, producing excess lipid peroxides that cannot be cleared by an inactivated antioxidant defense system, such as the Xc-GSH-GPX4 axis. This results in cell membrane damage and ultimately leads to ferroptosis (Yao et al., 2021). Morphologically, genetically, and biochemically, ferroptosis is distinct from traditional forms of cell death, such as apoptosis, necrosis, and autophagy (Xu S. et al., 2021). Morphologically, cells undergoing ferroptosis exhibit shrunken mitochondria with increased membrane density, ruptured outer membranes, and reduced or absent cristae, impairing mitochondrial function. Genetically, ferroptosis involves altered expression of genes regulating iron homeostasis and lipid oxidation. For instance, activating transcription factor 3 (ATF3) binds to the solute carrier family 7 member 11 (SLC7A11) promoter, suppressing SLC7A11 expression, which reduces levels of glutathione (GSH) and glutathione peroxidase 4 (GPX4) and hinders the reduction of lipid peroxides. Concurrently, the activity of ACSL4 (acyl-CoA synthetase long-chain family member 4), a driver of ferroptosis, is increased, promoting fatty acid metabolism and exacerbating lipid oxidation in cell membranes (Tang D. et al., 2021). Additionally, the expression of ferritin heavy polypeptide 1 (FTH1), an iron-storage protein, decreases, while the tumor suppressor gene p53 accelerates ferroptosis by transcriptionally inhibiting SLC7A11 expression (Yang et al., 2022). Biochemically, ferroptosis involves two main components: iron accumulation and lipid peroxidation. Excess iron generates reactive oxygen species (ROS) through the Fenton reaction, activating the mitogen-activated protein kinase (MAPK) pathway, reducing cystine uptake, depleting intracellular GSH, inhibiting the cystine/glutamate antiporter System XC-, and increasing nicotinamide adenine dinucleotide phosphate (NADPH) oxidation. Meanwhile, ROS oxidizes PUFAs in membrane phospholipids to lipid peroxides, leading to cell rupture and death (Figure 3).

Ferroptosis is associated with the occurrence and progression of multiple diseases, including cardiovascular diseases, kidney diseases, gastrointestinal disorders, cancers, and various neurodegenerative diseases (Wu et al., 2021; Martin-Sanchez et al., 2020; Xu et al., 2020; Qiu et al., 2020). Recent studies indicate that ferroptosis plays a significant role in the pathogenesis of depression. Proteomic analysis of hippocampal tissue from chronic unpredictable mild stress (CUMS) mice reveals 47 differentially expressed proteins with biological functions when compared to control groups. Among these, receptor-interacting protein 3 (RIP3), phosphorylated mixed lineage kinase domain-like protein (p-MLKL), and ferritin light chain 1 (FTL1) exhibit significant changes in expression and are associated with ferroptosis (Cao et al., 2021). Furthermore, ferroptosis-related genes, such as ALOX15B (Arachidonate-15-Lipoxygenase, Type B) and RPLP0 (ribosomal protein lateral stalk subunit P0), have been identified as potential biomarkers for diagnosing depression (Chen et al., 2023). Inhibiting ferroptosis has been shown to alleviate depressive symptoms, suggesting that ferroptosis may represent a novel therapeutic target for depression with broad research prospects (Xu et al., 2022; Wang L. et al., 2023).

Therefore, this review focuses on animal models of depression with a focus on ferroptosis, systematically summarizing the underlying mechanisms of the disease and existing therapeutic strategies, with the aim of providing a foundation for future in-depth investigations and the development of novel therapeutic approaches.

## 2 The relationship between GPX4-mediated signaling pathways of ferroptosis and depression

GPX4, a member of the glutathione peroxidase (GPX) superfamily, serves as a key regulator of ferroptosis. GPX4 is a lipid-dependent membrane repair enzyme that exists in several isoforms (cGPX4, GI-GPX, P-GPX, pH-GPX). Among these isoforms, GPX4 is the only enzyme capable of reducing esterified oxidized fatty acids and cholesterol hydroperoxides (Conrad and Friedmann Angeli, 2015). GPX4 plays a critical role in maintaining mitochondrial integrity and protecting ATP production, being closely related to cellular antioxidant functions (Yagi et al., 1998). GPX4 reduces phospholipid hydroperoxide (PL-OOH) to lipid alcohol (PL-OH), thereby decreasing membrane damage caused by uncontrolled lipid peroxidation.

In the absence of GPX4, the balance between the formation and reduction of lipid hydroperoxides (LOOH) is disrupted, leading to an imbalance in the cellular redox state. Unsaturated fatty acids generate highly reactive lipid peroxides (LPO) through free radical reactions. As the sole intracellular LPO-reducing enzyme, the lack of GPX4 results in excessive accumulation of LPO, destabilizing biological membranes, exacerbating oxidative stress, and ultimately promoting ferroptosis (Ursini and Maiorino, 2020). When excessive iron accumulates in the mitochondria, Fe^2+^ generates a large amount of hydroxyl radicals (·OH) through the Fenton reaction, resulting in mitochondrial dysfunction and structural damage (Abe et al., 2022). Increasing GPX4 expression can prevent oxidative stress-induced loss of mitochondrial membrane potential, enhance ATP production, and protect mitochondria from oxidative damage (Liang et al., 2007). The signaling pathways through which ferroptosis affects depression are predominantly mediated by GPX4.

### 2.1 PEBP1-GPX4 signaling pathway

Phosphatidylethanolamine-binding protein 1 (PEBP1) binds to 15-lipoxygenase (15-LO) to form the PEBP1/15-LO complex, which serves as a primary regulator of ferroptosis (Wenzel et al., 2017). During ferroptosis, the PEBP1/15-LO complex selectively reacts with one of the polyunsaturated fatty acids (PUFA), ethanolamine plasmalogen of eicosatetraenoic acid (ETE-PE), producing the peroxide 15-hydroperoxyeicosatetraenoyl-phosphatidylethanolamine (15-HpETE-PE) that induces cellular ferroptosis (Kagan et al., 2017). Studies show that the levels of GPX4 mRNA and protein are significantly reduced in the hippocampus of CUMS mice, while the expression of PEBP1 is upregulated and the levels of Fe and Fe^2+^ are notably increased. These findings suggest that iron accumulation is likely associated with CUMS-induced depression. Following treatment with antidepressants, not only do the depressive symptoms in mice improve significantly, but GPX4 expression also increases markedly, total iron content decreases, and PEBP1 levels drop, leading to improved cellular function (Jiao et al., 2021; Geng et al., 2022). Additionally, injecting adipose-derived mesenchymal stem cells (ADSC) into chronic mild stress (CMS)-induced depressed mice results in ameliorated depressive behaviors and elevated levels of PEBP1 and GPX4 in the hippocampus, indicating that PEBP1 may serve as a bridge linking the pathogenesis of depression with ferroptosis. The antidepressant effects of ADSC may be related to the activation of the PEBP1-GPX4 axis.

### 2.2 IRF1/SLC7A11/GPX4 signaling pathway

The ferroptosis-related IRF1/SLC7A11/GPX4 signaling pathway also plays an important role in depression (Tian et al., 2013). SLC7A11 functions as a reverse transporter for glutamate (Glu), participating in the release of glutamate and promoting the synthesis of GSH, which is a crucial cofactor for the activity of GPX4 and an important target for ferroptosis (Bassi et al., 2001). SLC7A11 exhibits negative feedback regulation on ferroptosis; its downregulation leads to decreased GSH synthesis, inhibiting GPX4 activity and weakening the cell’s antioxidant capacity, thereby increasing the risk of ferroptosis (Koppula et al., 2021). IRF1 (interferon regulatory factor 1), a member of the IRF family, plays significant roles in tumor suppression and immune regulation (Feng et al., 2021). Enhanced expression of IRF1 inhibits GPX4 activity, exacerbates lipid peroxidation, and promotes ferroptosis (Xu X. et al., 2021). Yao constructed a mutant recombinant luciferase reporter gene (pGL3.0-GPX4-M1) based on pGL3.0-GPX4-P1, discovering that mutations in pGL3.0-GPX4-P1 block IRF1-mediated fluorescence decay. They propose that under the stimulation of the pro-inflammatory cytokine TNF-*α*, IRF1 can directly bind to the GPX4 promoter region (−1,498 to −1,239), affecting GPX4 transcription. ChIP-PCR assays further validate this notion (Zhang Y. et al., 2022), suggesting that reducing IRF1 levels can alleviate the transcriptional repression of GPX4, thereby inhibiting ferroptosis.

### 2.3 FTH1/TFR1/GPX4 signaling pathway

FTH1 protein is a critical component of ferritin, functioning to reduce intracellular free iron by storing it (Kong et al., 2021), thereby regulating cellular iron ion levels and playing an important role in ferroptosis. Transferrin receptor 1 (TFR1) forms a complex with its ligand transferrin (TF), jointly regulating cellular iron absorption. Overexpression of TFR1 increases iron uptake, enhancing cellular sensitivity to ferroptosis, while knockdown of TFR1 improves erastin-induced ferroptosis. TFR1 can serve as a specific biomarker for ferroptosis, positively correlating with its occurrence (Lan et al., 2023; Chen et al., 2023). During ferroptosis, levels of FTH1 and GPX4 decrease while TFR1 levels increase. TFR1, which is localized in the Golgi apparatus, translocates to the cytoplasmic membrane and facilitates the transport of extracellular ferritin into the cell, accelerating the ferroptosis process (Tang L. J. et al., 2021; Lu et al., 2020; Zhao et al., 2022; Yang and Stockwell, 2008). Zhang M. et al. (2022) observed the activation of ferroptosis in the nucleus of the nucleus accumbens of chronic restraint stress (CRS)-induced depressed rats (Proulx et al., 2014; Lawson et al., 2017). After treatment with amiodarone, they found significant recovery in the previously reduced expressions of FTH1 and GPX4, alongside a marked decrease in Trf1 expression, resulting in rapid alleviation of depressive-like symptoms. This suggests that regulating the FTH1/TFR1/GPX4 signaling pathway may inhibit ferroptosis as a potential treatment for depression.

### 2.4 ALKBH5-PRMT2-*β*-catenin-GPX4 signaling pathway

The ALKBH5-PRMT2-β-catenin-GPX4 axis is believed to play an important role in promoting LPS (lipopolysaccharide)-induced ferroptosis in microglial cells, providing new insights into the pathogenesis of depression. ALKBH5-mediated N6-methyladenosine (m6A) modification enhances the stability of PRMT2 mRNA; abnormal m6A modifications are often associated with depressive-like behaviors. It has been reported that β-catenin can reduce the production of lipid peroxides, and the β-catenin/TCF4 transcription complex can directly bind to the promoter region of GPX4 to activate its transcription, thereby inhibiting ferroptosis (Wang Y. et al., 2022). PRMT2 (protein arginine methyltransferase 2) promotes the arginine methylation of β-catenin, leading to the degradation of β-catenin protein and subsequently inhibiting GPX4 transcription. In the LPS-induced depression model, the demethylation function of m6A-modified PRMT2 mRNA is inhibited, resulting in increased expression of PRMT2 in BV2 cells and the hippocampus, while ALKBH5, SLC7A11, and GPX4 expressions decrease, facilitating ferroptosis. When ALKBH5 is overexpressed, PRMT2 expression is inhibited, alleviating ferroptosis. This suggests that ALKBH5 may alleviate ferroptosis in BV2 cells and improve depressive symptoms by inhibiting PRMT2 through the β-catenin-GPX4 axis (Mao et al., 2024).

In summary, various proteins interact with GPX4 to play significant roles in the process of ferroptosis. GPX4-mediated ferroptosis-related signaling pathways represent promising targets for the treatment of depression (Zhang et al., 2024).

## 3 The relationship between Nrf2-related signaling pathways in ferroptosis mechanisms and depression

Nrf2 (nuclear factor erythroid 2-related factor 2) is a critical transcription factor that regulates cellular oxidative stress responses. It mitigates cell damage caused by reactive oxygen species (ROS) and electrophiles by modulating the expression of a range of antioxidant genes, serving as a key regulator of the endogenous antioxidant defense system (Abdalkader et al., 2018). Under non-stress conditions, low levels of Nrf2 remain in an inactive state primarily due to proteasomal degradation mediated by Keap1 (Sun et al., 2016; Fan et al., 2017). However, when intracellular ROS levels increase, Nrf2 is released from its binding site on Keap1, rapidly translocated to the nucleus. There, it binds to antioxidant response elements (ARE) in the promoter regions of target genes, promoting the transcription of various antioxidant enzymes, regulating ROS production, enhancing iron storage, reducing cellular iron absorption, and limiting iron generation. This process balances the redox state within the body, maintaining intracellular homeostasis and lowering the risk of ferroptosis (Song and Long, 2020). Many enzymes that prevent lipid peroxidation and inhibit ferroptosis are target genes of Nrf2, including FTH1, GSH, and SLC7A11. Additionally, ferroptosis inducers such as RAS-selective lethal 3 (RSL-3) and Erastin, which inhibit GPX4 activity and the cysteine/glutamate transport system (xC-/xCT), are also downstream targets of Nrf2 (Dodson et al., 2019; Wang X. et al., 2022). Nrf2 not only participates in the regulation of oxidative homeostasis but is also involved in processes related to neuroinflammation and mitochondrial dysfunction, both closely linked to the development of depression (Zuo et al., 2022). Studies reveal a decrease in Nrf2 protein expression in the prefrontal cortex (PFC) of patients with major depressive disorder (MDD), while Nrf2 knockout mice exhibit depressive-like behaviors. The use of the Nrf2 activator oltipraz effectively inhibits the expression of transferrin receptor (TFR) and divalent metal transporter (DMT1), which in turn suppresses ferroptosis and alleviates depressive symptoms (Zeng et al., 2023).

### 3.1 BDNF/Nrf2 signaling pathway

Brain-derived neurotrophic factor (BDNF) is closely associated with clinical changes in depression, as indicated by meta-analyses linking BDNF levels to depressive disorders (Brunoni et al., 2008). Decreased BDNF levels in the hippocampus lead to an increase in the frequency of depressive behaviors, with fluctuations observed throughout the progression of the disease (Martinowich et al., 2007). In the brains of MDD patients, BDNF expression is reduced, resulting in neurodevelopmental abnormalities and a significant decrease in neuron numbers in the cerebral cortex, hippocampus, and dorsal thalamus. Magnetic resonance imaging (MRI) also reveals damage to gray and white matter, indicating that BDNF may serve as a potential biomarker for depression (Peng et al., 2018; Belleau et al., 2019). Furthermore, low levels of BDNF inhibit the effective translocation of Nrf2 to the nucleus, impairing Nrf2 pathway functionality and limiting the activation of antioxidant mechanisms, ultimately resulting in persistent oxidative stress (Bouvier et al., 2017). This state of oxidative stress not only contributes to the onset of depressive behaviors but also promotes the progression of ferroptosis. Electroconvulsive therapy (ECT) is one of the effective methods for treating MDD by suppressing ferroptosis in hippocampal neurons. Following ECT treatment in CUMS rats, an increase in hippocampal neuron numbers, along with elevated expression of GPX4 and FTH1 and reduced expression of ACSL4, is observed, along with increased protein levels of BDNF and Nrf2, thereby inhibiting ferroptosis and alleviating depressive symptoms. However, administration of the Nrf2 inhibitor all-trans retinoic acid (ATRA) results in downregulation of SLC7A11, GPX4, and FTH1, along with increased levels of Fe^2+^ and MDA, indicating the loss of ECT’s inhibitory effect on ferroptosis in hippocampal neurons. This suggests that the inhibitory effect of ECT on ferroptosis depends on BDNF-mediated activation of Nrf2 (Li X. et al., 2023). Moreover, studies have shown that isochlorogenic acid A (ICAA) can alleviate lead-induced anxiety-like behaviors. ICAA enhances levels of Nrf2, HO-1, and BDNF in the brain by suppressing the expression of TNF-*α* and IL-6, thus activating the BDNF/Nrf2/GPX4 axis. This effectively improves neuroinflammation and inhibits ferroptosis, reducing anxiety-like behaviors (Guo et al., 2024). These findings indicate that the BDNF/Nrf2 pathway is a promising target for treating depression and anxiety disorders.

### 3.2 Sirt1, Sirt6/Nrf2/HO-1/GPX4 signaling pathway

The Sirt1/Nrf2/HO-1/GPX4 axis exhibits antidepressant and anxiolytic effects (Dang et al., 2022). Sirt1 (silent information regulator 1) is an NAD^+^-dependent deacetylase involved in regulating cellular metabolism, oxidative stress, and inflammatory responses. It protects neurons by improving mitochondrial biogenesis and counteracting apoptosis. Studies demonstrate that modulating the Sirt1-mediated signaling pathway can reduce ferroptosis in hippocampal neurons (Wang et al., 2023a). Heme oxygenase-1 (HO-1), one of two different HO isoforms in mammals, acts as a critical downstream protein of Nrf2. The cytoprotective role of HO-1 during oxidative stress relies on its interaction with Nrf2 (Biswas et al., 2014); a reduction in Nrf2 significantly decreases HO-1 expression. Expression levels of Sirt1, Nrf2, HO-1, and GPX4 are markedly reduced in mice subjected to chronic social defeat stress (CSDS), leading to varying degrees of mitochondrial and neuronal damage. Edaravone (EDA), a free radical scavenger with antioxidant and anti-inflammatory properties, increases expression levels of Sirt1, Nrf2, HO-1, and GPX4 in CSDS rats, inhibiting ferroptosis and alleviating neuronal damage, which consequently improves depressive symptoms. After administering the Sirt1 inhibitor EX527 to CSDS mice, levels of Nrf2, HO-1, and GPX4 decrease, suggesting that Sirt1 may enhance antidepressant effects via the Nrf2/HO-1/GPX4 pathway. Further inhibition of Nrf2 does not significantly alter Sirt1 expression, indicating that Nrf2 is a downstream target of Sirt1. Lastly, blocking GPX4 abolishes the antidepressant effects of EDA, confirming the regulatory role of the Sirt1/Nrf2/HO-1/GPX4 signaling pathway in ferroptosis, with GPX4 serving as a crucial component in the treatment of depression and anxiety via ferroptosis mechanisms (Dang et al., 2022). Melatonin (MLT), an endogenous hormone produced and secreted by the pineal gland, plays an important role in regulating circadian rhythms and is closely linked to various mechanisms associated with neurotransmitters, their receptors, and the release of inflammatory factors in depression (Hickie and Rogers, 2011; Tonon et al., 2021). Research indicates that MLT may inhibit ferroptosis through the Sirt6/Nrf2/HO-1 pathway, thus exerting antidepressant effects. Sirt6, a member of the NAD^+^-dependent deacetylase family, produces antidepressant effects when inhibited (Hu et al., 2023), mediating MLT’s suppression of cellular ferroptosis. Treatment of LPS-induced depressive mice with MLT results in decreased concentrations of ROS, MDA, and Fe^2+^, alongside reduced ACSL4 levels and elevated SOD, GSH, GPX4, and FSP1 levels, leading to improved depressive symptoms. In these mice, reduced expression of Sirt6 is accompanied by significant activation of Nrf2/HO-1 as downstream targets of Sirt6. The use of the Nrf2 inhibitor ML385 results in the loss of MLT’s inhibitory effects on ferroptosis, highlighting the essential role of the Sirt6/Nrf2/HO-1 pathway in MLT’s inhibition of ferroptosis and improvement of depressive symptoms (Su et al., 2024).

Currently, research on the role of Nrf2 in the ferroptosis mechanisms associated with depression is limited, and its specific pathways remain unclear. Clinical applications of Nrf2 activators, such as fumaric acid, are currently under exploration with promising efficacy. Therefore, investigating the Nrf2-mediated ferroptosis mechanisms related to depression holds significant potential and may provide valuable research directions for the treatment of depression.

## 4 The relationship between other ferroptosis-related regulators and depression

Research indicates that the onset of depression is influenced by multiple factors, including genetic, epigenetic, and environmental components, and is closely associated with the expression of small non-coding RNAs (sncRNAs), such as miRNAs and tsRNAs. Changes in the expression levels of non-coding small RNAs have been observed in various regions of the brains of patients with depression, including miR-484-5p, miR-26b-5p, miR-30d-5p, and miR-197-3p in the BA9 region; miR-34c-5p, miR-139-5p, and miR-124-3p in the BA44 region; and miR-323a-3p in the anterior cingulate cortex (ACC) (Żurawek and Turecki, 2021). Currently, novel tsRNAs derived from tRNA are receiving increased attention as potential targets for early diagnosis and treatment of diseases such as Alzheimer’s disease and cerebral hemorrhage. One study found that in CUMS mice, the expression of tsRNA-3029b was upregulated, resulting in a significant increase in reactive oxygen species (ROS) levels in the blood and a decrease in the content of SLC7A11 and GPX4. Conversely, silencing tsRNA-3029b reversed these results and inhibited ferroptosis. In cortisol (CORT)-induced impaired neurons, silencing tsRNA-3029b promoted neuronal regeneration. These findings suggest that tsRNA-3029b serves as a critical tsRNA that inhibits neuronal regeneration, promotes ferroptosis, and contributes to depressive symptoms, potentially serving as a therapeutic target for CUMS-induced depression (Li E. et al., 2023). Furthermore, long non-coding RNA taurine up-regulated gene 1 (TUG1) induces ferroptosis in hippocampal neurons by promoting the ubiquitin-mediated degradation of dual specificity phosphatase 14 (DUSP14), thereby inhibiting GPX4 levels. This mechanism is associated with depressive-like behaviors and holds significant clinical implications for diagnosing major depressive disorder (MDD) (Mei et al., 2024). P53, a key tumor suppressor gene, not only inhibits cancer by regulating the cell cycle and inducing apoptosis but also modulates cellular metabolism, autophagy, and oxidative status, participating in various complex physiological and pathological processes. P53 promotes ferroptosis by reducing SLC7A11 expression and inhibiting GPX4 activity, leading to iron accumulation and increased ROS levels (Brady and Attardi, 2010). Di Huang Yin Zi (DHYZ) is a traditional Chinese herbal formula composed of multiple herbs, including Rehmannia glutinosa, *Cornus officinalis*, and Dendrobium nobile, and it exhibits promising therapeutic effects in preventing and treating neurological disorders. Research has demonstrated that DHYZ extract can enhance P53 ubiquitination and degradation in rats with post-stroke depression, leading to the upregulation of GPX4 and SLC7A11 protein expression. This suggests that DHYZ may alleviate depressive symptoms while inhibiting ferroptosis by modulating the P53/SLC7A11/GPX4 signaling pathway in brain tissue, consequently reducing the levels of reactive oxygen species (ROS) and malondialdehyde (MDA) (Yang et al., 2024). However, due to the multi-component synergistic nature of traditional Chinese herbal formulas, the specific active components responsible for these effects remain to be further investigated.

Recent studies suggest that the regulation of gene expression may be a crucial mechanism underlying the occurrence of ferroptosis in depression, particularly highlighting the significant relationship of stress-related miRNAs as potential biomarkers for depression and antidepressant treatment (Chen et al., 2024). Additionally, regulatory factors such as TUG1 and P53 play important roles in the ferroptosis mechanism of depression, but further research is needed to validate these findings.

## 5 Depression treatment under the theory of ferroptosis

### 5.1 Antidepressant compounds

Currently, the treatment of depression includes commonly used medications such as selective serotonin reuptake inhibitors (SSRIs) and norepinephrine reuptake inhibitors, as well as certain compounds and traditional Chinese medicine therapies that exhibit antidepressant effects (Kent, 2000). Hydrogen sulfide (H_2_S) is a gaseous signaling molecule involved in regulating various physiological and pathological processes in the central nervous system, demonstrating antidepressant properties (Kang et al., 2021). Patients with type 1 diabetes (DM) are particularly susceptible to anxiety and depression due to environmental and personal factors. Research shows that H_2_S not only alleviates depressive behaviors in DM mice but also reduces iron deposition in the prefrontal cortex (PFC) of these mice, increases the expression of GSH, GPX4, and SLC7A11, and decreases levels of MDA and ROS in the blood, thereby inhibiting ferroptosis. Additionally, H_2_S inhibits the activation of microglia and the release of pro-inflammatory cytokines, enhances Sirt6 expression, and promotes the interaction between Sirt6 and histone H3 lysine 9 (H3K9ac), ultimately reducing H3K9ac protein and Notch1 receptor expression to alleviate inflammatory responses. These findings suggest that H_2_S may mitigate depressive-like behaviors in DM animals by reducing inflammation and inhibiting ferroptosis (Wang et al., 2021; Tang et al., 2015).

Sestrin2 (SESN2) is a protein that protects cells from stress and age-related damage. The promotion of SESN2 protein expression in CUMS mice significantly improves depressive symptoms while reducing levels of pro-inflammatory cytokines TNF-*α*, IL-6, IL-1β, and Iba-1 in the hippocampus, alleviating microglial activation and inflammation. SESN2 also decreases iron deposition and reverses the elevated expression of ACSL4 and TFR1 while enhancing the expression of SCL7A11 and GPX4, thereby inhibiting ferroptosis. This effect has also been observed in LPS-induced BV-2 cells in mice (Ma et al., 2024).

Eicosapentaenoic acid (EPA) and docosahexaenoic acid (DHA) are bioactive compounds found in fish oil. Although EPA is present in smaller amounts in the brain, its antidepressant and anti-inflammatory capabilities surpass those of DHA (Peng et al., 2020). Wang utilized a pentylene tetrazole (PTZ)-induced depression mouse model, revealing that EPA and DHA alleviate depressive behaviors in mice by inhibiting inflammation and ferroptosis. In the PTZ model, total iron and Fe^2+^ levels increase, with significant upregulation of iron regulatory proteins IRP1, IRP2, and TfR1, along with a decrease in ferroportin 1 (FPN1) and FTH1, and reduced GSH levels accompanied by increased MDA content. The expression of GPX4, xCT, HO-1, and p-Nrf2 proteins decreases, leading to heightened ferroptosis of hippocampal neurons. Treatment with EPA and DHA reverses these changes, with the EPA group exhibiting higher levels of p-Nrf2, xCT, and HO-1 expression, as well as reduced iron accumulation. Moreover, EPA and DHA can inhibit the expression of pro-IL-1β, IL-1β, and TNF-α, with EPA specifically reducing the binding of NLRP3 and apoptosis-associated speck-like protein containing a CARD (ASC), thereby inhibiting NLRP3 inflammasome activation. Therefore, it is concluded that EPA and DHA effectively alleviate cellular inflammatory responses, inhibit ferroptosis, and ultimately improve depressive behaviors in mice (Wang X. et al., 2022).

### 5.2 Therapies derived from traditional Chinese medicine

Traditional Chinese medicine (TCM) has a history spanning several millennia and offers unique advantages in the treatment of depression. Various extracts from traditional Chinese herbs demonstrate significant efficacy in alleviating depressive symptoms. Gallic acid (3,4,5-trihydroxybenzoic acid), found in the galla chinensis, exhibits anti-inflammatory and antioxidant properties. Yang applied gallic acid in a treatment regimen for rats with chronic constriction injury (CCI) combined with chronic unpredictable mild stress (CUMS). The results indicated a reduction in levels of ROS, MDA, and iron in the blood, an increase in the expression of GSH and GPX4, and alleviation of oxidative stress-induced mitochondrial damage in the spinal cord. Furthermore, ferroptosis was inhibited, depressive symptoms were alleviated, and the expression of the P2X7 receptor, which is involved in depression, was suppressed. This suggests that gallic acid’s alleviation of depressive-like behavior in rats by inhibiting spinal microglia ferroptosis may be achieved through modulation of the P2X7-ROS signaling pathway (Yang et al., 2023). Furthermore, as an iron chelator, gallic acid’s iron-chelating ability itself may also contribute to ferroptosis inhibition (Strlic et al., 2002).

The use of Bupleurum in the treatment of depression has a history of over a thousand years. Studies reveal that the active component extracted from Bupleurum, Sasycosaponin B2 (SSB2), significantly improves depressive symptoms by reducing levels of ROS and Fe^2+^ in microglia, enhancing the transcription levels of GSH, SLC7A11, FTH, GPX4, and Nrf2, while downregulating ACSL4 and Tfr1 to inhibit lipid peroxidation and cellular ferroptosis. Additionally, it modulates the TLR4/NF-κB pathway to inhibit microglial activation, reduce neuroinflammation, and ameliorate cellular damage, ultimately improving depressive behaviors in mice (Wang et al., 2023b).

*Lycium barbarum* polysaccharides (LbGp), derived from the goji berry, exhibit protective effects on hippocampal neurons in depressed rats and exert antidepressant and anxiolytic effects in psychiatric disorders (Soytürk et al., 2023). Dai et al. (2023) demonstrated that LbGp enhances the activity of superoxide dismutase (SOD), reduces MDA and 4-hydroxynonenal (4-HNE) levels, and increases GPX4 expression, thereby alleviating oxidative stress and lipid peroxidation in the medial prefrontal cortex (mPFC). This inhibition of ferroptosis, resulting from GPX4 knockout, effectively mitigates anxiety and depressive behaviors under chronic restraint stress.

Chrysophanol (CNS), an antioxidant flavonoid extract from honeysuckle, shows potential in treating LPS-induced depressive models. After CNS treatment, a decrease in intracellular Fe^2+^ and MDA levels (Stockwell, 2022) and a reduction in IRF1 expression were observed, while SLC7A11, GPX4, and GSH levels increased, leading to notable improvements in depressive-like behaviors in mice. Furthermore, transcriptomic analysis indicates that CNS may inhibit LPS-induced cellular ferroptosis through the IRF1/SLC7A11/GPX4 pathway, effectively improving depressive states.

Quercetin has also been shown to alleviate breast cancer-related depression (BCRD). Zhu et al. (2024) investigated the effects of quercetin on the expression of genes associated with ferroptosis and lipid metabolism in neurons of BCRD mice. Through PPI network and MCODE clustering analysis, key lipid metabolism genes were identified. Post-treatment with quercetin, significant reductions were noted in the expression of IL-6, AKT1, IL-1β, and PTGS2, particularly in prostaglandin-endoperoxide synthase 2 (PTGS2) expression, which correlates with increased total Fe, Fe^2+^, MDA, and ROS levels. Moreover, inhibition of PTGS2 expression by quercetin enhanced the survival rate of hippocampal neurons and upregulated levels of 5-HT, DA, and NE in BCRD mice, alleviating depressive symptoms. This indicates that quercetin targets PTGS2 to reduce ferroptosis in neurons, thereby mitigating depressive symptoms.

Acupuncture, regarded as one of the most prominent treasures of traditional Chinese medicine, also plays a significant role in treating depression. Research shows that acupuncture at the Fengfu (GV16) and Shangxing (GV23) points in CUMS mice results in decreased levels of ROS and MDA in serum, while enhancing the expression of SOD, GSH, and GSH-PX. Furthermore, mRNA levels of Sirt1, Nrf2, HO-1, and GPX4 in the hippocampus increase post-acupuncture. Additionally, activation of microglia and astrocytes is significantly reduced, and the expression of inflammatory factors IL-1β and TNF-*α* decreases. It is suggested that acupuncture may modulate ROS levels in the brain, mitigate cellular damage, alleviate neuroinflammation, and inhibit ferroptosis through the stimulation of the Sirt1/Nrf2/HO-1/GPX4 pathway, ultimately improving depressive symptoms (Shen et al., 2024).

## 6 Summary and outlook

This article summarizes the signaling pathways related to ferroptosis in depression and discusses how regulatory factors, such as non-coding RNAs, influence depression by modulating ferroptosis. Additionally, it introduces potential medications and methods for treating depression based on the principles of ferroptosis. The findings suggest that inhibiting ferroptosis can significantly alleviate depressive symptoms, positioning it as a promising avenue for future research in depression treatment.

Moreover, many currently developed therapeutic agents originate from active compounds in traditional Chinese medicine (TCM) formulas or herbal extracts, which are abundant in raw materials and exhibit significant efficacy. Acupuncture, characterized by its convenience and low cost, is gradually gaining acceptance and can be combined with pharmacological treatments in clinical settings for enhanced effectiveness. Therefore, alongside the intensified research and development of Western pharmaceuticals, the rational utilization of traditional Chinese medical approaches should also be emphasized.

Additionally, current research remains limited in scope. Questions persist regarding whether ferroptosis regulates neurotransmitters and their receptors, subsequently affecting neuroendocrine function and the development of depression. Furthermore, studies investigating TCM treatments for depression based on the ferroptosis framework are scarce, primarily focusing on depressive animal models, with a lack of supportive clinical trial results. Future research should prioritize addressing these issues to further elucidate the role of ferroptosis in depression and provide targets for the development of new antidepressant medications.

## References

[ref1] Abdalkader M. Lampinen R. Kanninen K. M. Malm T. M. Liddell J. R. (2018). Targeting Nrf2 to suppress ferroptosis and mitochondrial dysfunction in neurodegeneration. Front. Neurosci. 12:466. doi: 10.3389/fnins.2018.00466, PMID: 30042655 PMC6048292

[ref2] Abe C. Miyazawa T. Miyazawa T. (2022). Current use of Fenton reaction in drugs and food. Molecules 27:5451. doi: 10.3390/molecules27175451, PMID: 36080218 PMC9457891

[ref3] Bair M. J. Robinson R. L. Katon W. Kroenke K. (2003). Depression and pain comorbidity: a literature review. Arch. Intern. Med. 163, 2433–2445. doi: 10.1001/archinte.163.20.2433, PMID: 14609780

[ref4] Bassi M. Gasol E. Manzoni M. Pineda M. Riboni M. Martín R.. (2001). Identification and characterisation of human xCT that co-expresses, with 4F2 heavy chain, the amino acid transport activity system xc^—^. Pflugers Arch. 442, 286–296. doi: 10.1007/s004240100537, PMID: 11417227

[ref5] Belleau E. L. Treadway M. T. Pizzagalli D. A. (2019). The impact of stress and major depressive disorder on hippocampal and medial prefrontal cortex morphology. Biol. Psychiatry 85, 443–453. doi: 10.1016/j.biopsych.2018.09.031, PMID: 30470559 PMC6380948

[ref6] Bhatt S. Nagappa A. N. Patil C. R. (2020). Role of oxidative stress in depression. Drug Discov. Today 25, 1270–1276. doi: 10.1016/j.drudis.2020.05.001, PMID: 32404275

[ref7] Biswas C. Shah N. Muthu M. la P. Fernando A. P. Sengupta S.. (2014). Nuclear heme oxygenase-1 (HO-1) modulates subcellular distribution and activation of Nrf2, impacting metabolic and anti-oxidant defenses. J. Biol. Chem. 289, 26882–26894. doi: 10.1074/jbc.M114.567685, PMID: 25107906 PMC4175329

[ref8] Bouvier E. Brouillard F. Molet J. Claverie D. Cabungcal J. H. Cresto N.. (2017). Nrf2-dependent persistent oxidative stress results in stress-induced vulnerability to depression. Mol. Psychiatry 22, 1701–1713. doi: 10.1038/mp.2016.144, PMID: 27646262

[ref9] Brady C. A. Attardi L. D. (2010). p53 at a glance. J. Cell Sci. 123, 2527–2532. doi: 10.1242/jcs.064501, PMID: 20940128 PMC2912460

[ref10] Brunoni A. R. Lopes M. Fregni F. (2008). A systematic review and meta-analysis of clinical studies on major depression and BDNF levels: implications for the role of neuroplasticity in depression. Int. J. Neuropsychopharmacol. 11, 1169–1180. doi: 10.1017/S1461145708009309, PMID: 18752720

[ref11] Cao H. Zuo C. Huang Y. Zhu L. Zhao J. Yang Y.. (2021). Hippocampal proteomic analysis reveals activation of necroptosis and ferroptosis in a mouse model of chronic unpredictable mild stress-induced depression. Behav. Brain Res. 407:113261. doi: 10.1016/j.bbr.2021.113261, PMID: 33775778

[ref12] Chen J. Jiang X. Gao X. Wu W. Gu Z. Yin G.. (2023). Ferroptosis-related genes as diagnostic markers for major depressive disorder and their correlations with immune infiltration. Front. Med. 10:1215180. doi: 10.3389/fmed.2023.1215180, PMID: 37942417 PMC10627962

[ref13] Chen L. Ma Y. Ma X. Liu L. Jv X. Li A.. (2023). TFEB regulates cellular labile iron and prevents ferroptosis in a TfR1-dependent manner. Free Radic. Biol. Med. 208, 445–457. doi: 10.1016/j.freeradbiomed.2023.09.004, PMID: 37683766

[ref14] Chen H. Wu J. Zhu X. Ma Y. Li Z. Lu L.. (2024). Manganese-induced miR-125b-2-3p promotes anxiety-like behavior via TFR1-mediated ferroptosis. Environ. Pollut. 344:123255. doi: 10.1016/j.envpol.2023.123255, PMID: 38159631

[ref15] Conrad M. Friedmann Angeli J. P. (2015). Glutathione peroxidase 4 (Gpx4) and ferroptosis: what's so special about it? Mol. Cell. Oncol. 2:e995047. doi: 10.4161/23723556.2014.995047, PMID: 27308484 PMC4905320

[ref16] Dai Y. Guo J. Zhang B. Chen J. Ou H. He R. R.. (2023). *Lycium barbarum* (wolfberry) glycopeptide prevents stress-induced anxiety disorders by regulating oxidative stress and ferroptosis in the medial prefrontal cortex. Phytomedicine 116:154864. doi: 10.1016/j.phymed.2023.154864, PMID: 37182278

[ref17] Dang R. Wang M. Li X. Wang H. Liu L. Wu Q.. (2022). Edaravone ameliorates depressive and anxiety-like behaviors via Sirt1/Nrf2/HO-1/Gpx4 pathway. J. Neuroinflammation 19:41. doi: 10.1186/s12974-022-02400-6, PMID: 35130906 PMC8822843

[ref18] Dixon S. J. Lemberg K. M. Lamprecht M. R. Skouta R. Zaitsev E. M. Gleason C. E.. (2012). Ferroptosis: an iron-dependent form of nonapoptotic cell death. Cell 149, 1060–1072. doi: 10.1016/j.cell.2012.03.042, PMID: 22632970 PMC3367386

[ref19] Dodson M. Castro-Portuguez R. Zhang D. D. (2019). NRF2 plays a critical role in mitigating lipid peroxidation and ferroptosis. Redox Biol. 23:101107. doi: 10.1016/j.redox.2019.101107, PMID: 30692038 PMC6859567

[ref20] Du L. Wu Y. Fan Z. Li Y. Guo X. Fang Z.. (2023). The role of ferroptosis in nervous system disorders. J. Integr. Neurosci. 22:19. doi: 10.31083/j.jin2201019, PMID: 36722234

[ref21] Fan Z. Wirth A. K. Chen D. Wruck C. J. Rauh M. Buchfelder M.. (2017). Nrf2-Keap1 pathway promotes cell proliferation and diminishes ferroptosis. Oncogenesis 6:e371. doi: 10.1038/oncsis.2017.65, PMID: 28805788 PMC5608917

[ref22] Feng H. Zhang Y. B. Gui J. F. Lemon S. M. Yamane D. (2021). Interferon regulatory factor 1 (IRF1) and anti-pathogen innate immune responses. PLoS Pathog. 17:e1009220. doi: 10.1371/journal.ppat.1009220, PMID: 33476326 PMC7819612

[ref23] Geng R. Dai M. Wang Y. Li H. B. Wang H. Huang X. (2022). Evaluation the therapeutic effect of adipose-derived mesenchymal stem cells on chronic mild stress by activating PEBP1-GPX4 Axis in Ferroptosis using qRT-PCR, fluorescence microscope and Iron determination analysis. J. Biomed. Nanotechnol. 18, 2828–2838. doi: 10.1166/jbn.2022.3475

[ref24] Guo J. T. Li H. Y. Cheng C. Shi J. X. Ruan H. N. Li J.. (2024). Isochlorogenic acid a ameliorated lead-induced anxiety-like behaviors in mice by inhibiting ferroptosis-mediated neuroinflammation via the BDNF/Nrf2/GPX4 pathways. Food Chem. Toxicol. 190:114814. doi: 10.1016/j.fct.2024.114814, PMID: 38876379

[ref25] Hickie I. B. Rogers N. L. (2011). Novel melatonin-based therapies: potential advances in the treatment of major depression. Lancet 378, 621–631. doi: 10.1016/S0140-6736(11)60095-0 21596429

[ref26] Hu K. Chen H. Gao Y. Hua R. Shi X. Li L.. (2023). Astrocytic SIRT6 is a potential anti-depression and anti-anxiety target. Prog. Neuro-Psychopharmacol. Biol. Psychiatry 123:110702. doi: 10.1016/j.pnpbp.2022.110702, PMID: 36565979

[ref27] Jiao H. Yang H. Yan Z. Chen J. Xu M. Jiang Y.. (2021). Traditional Chinese formula xiaoyaosan alleviates depressive-like behavior in CUMS mice by regulating PEBP1-GPX4-mediated ferroptosis in the hippocampus. Neuropsychiatr. Dis. Treat. 17, 1001–1019. doi: 10.2147/NDT.S302443, PMID: 33854318 PMC8039849

[ref28] Kagan V. E. Mao G. Qu F. Angeli J. P. F. Doll S. Croix C. S.. (2017). Oxidized arachidonic and adrenic PEs navigate cells to ferroptosis. Nat. Chem. Biol. 13, 81–90. doi: 10.1038/nchembio.2238, PMID: 27842066 PMC5506843

[ref29] Kang X. Jiang L. Lan F. Tang Y. Y. Zhang P. Zou W.. (2021). Hydrogen sulfide antagonizes sleep deprivation-induced depression-and anxiety-like behaviors by inhibiting neuroinflammation in a hippocampal Sirt1-dependent manner. Brain Res. Bull. 177, 194–202. doi: 10.1016/j.brainresbull.2021.10.002, PMID: 34624463

[ref30] Kent J. M. (2000). SNaRIs, NaSSAs, and NaRIs: new agents for the treatment of depression. Lancet 355, 911–918. doi: 10.1016/S0140-6736(99)11381-3, PMID: 10752718

[ref31] Kong N. Chen X. Feng J. Duan T. Liu S. Sun X.. (2021). Baicalin induces ferroptosis in bladder cancer cells by downregulating FTH1. Acta Pharm. Sin. B 11, 4045–4054. doi: 10.1016/j.apsb.2021.03.036, PMID: 35024325 PMC8727776

[ref32] Koppula P. Zhuang L. Gan B. (2021). Cystine transporter SLC7A11/xCT in cancer: ferroptosis, nutrient dependency, and cancer therapy. Protein Cell 12, 599–620. doi: 10.1007/s13238-020-00789-5, PMID: 33000412 PMC8310547

[ref33] Lan T. Hu L. Sun T. Wang X. Xiao Z. Shen D.. (2023). H3K9 trimethylation dictates neuronal ferroptosis through repressing Tfr1. J. Cereb. Blood Flow Metab. 43, 1365–1381. doi: 10.1177/0271678X231165653, PMID: 36960698 PMC10369154

[ref34] Lawson R. P. Nord C. L. Seymour B. Thomas D. L. Dayan P. Pilling S.. (2017). Disrupted habenula function in major depression. Mol. Psychiatry 22, 202–208. doi: 10.1038/mp.2016.81, PMID: 27240528 PMC5285459

[ref35] Li X. Hu J. Zang X. Xing J. Mo X. Hei Z.. (2023). Etomidate improves the antidepressant effect of electroconvulsive therapy by suppressing hippocampal neuronal ferroptosis via upregulating BDNF/Nrf2. Mol. Neurobiol. 60, 6584–6597. doi: 10.1007/s12035-023-03499-1, PMID: 37466875

[ref36] Li S. Sun Y. Song M. Song Y. Fang Y. Zhang Q.. (2021). NLRP3/caspase-1/GSDMD-mediated pyroptosis exerts a crucial role in astrocyte pathological injury in mouse model of depression. JCI Insight 6:e146852. doi: 10.1172/jci.insight.146852 34877938 PMC8675200

[ref37] Li E. Yin H. Su M. Li Q. Zhao Y. Zhang L.. (2023). Inhibition of ferroptosis alleviates chronic unpredictable mild stress-induced depression in mice via tsRNA-3029b. Brain Res. Bull. 204:110773. doi: 10.1016/j.brainresbull.2023.110773, PMID: 37793597

[ref38] Liang H. Van Remmen H. Frohlich V. Lechleiter J. Richardson A. Ran Q. (2007). Gpx4 protects mitochondrial ATP generation against oxidative damage. Biochem. Biophys. Res. Commun. 356, 893–898. doi: 10.1016/j.bbrc.2007.03.045, PMID: 17395155

[ref39] Lu J. Xu F. Lu H. (2020). LncRNA PVT1 regulates ferroptosis through miR-214-mediated TFR1 and p53. Life Sci. 260:118305. doi: 10.1016/j.lfs.2020.118305, PMID: 32827544

[ref40] Lucero-Prisno D. E. III Shomuyiwa D. O. Kouwenhoven M. B. N. Dorji T. Odey G. O. Miranda A. V.. (2023). Top 10 public health challenges to track in 2023: shifting focus beyond a global pandemic. Public Health Chall. 2:e86. doi: 10.1002/puh2.86 PMC12039733 40495865

[ref41] Ma X. Wang J. Quan Q. Zhang H. Tian Y. Wang L.. (2024). Sestrin2 attenuates depressive-like behaviors and neuroinflammation in CUMS mice through inhibiting ferroptosis. Neuroreport 35, 143–151. doi: 10.1097/WNR.0000000000001988, PMID: 38109473

[ref42] Mao L. You J. Xie M. Hu Y. Zhou Q. (2024). Arginine methylation of β-catenin induced by PRMT2 aggravates LPS-induced cognitive dysfunction and depression-like behaviors by promoting ferroptosis. Mol. Neurobiol. 61, 7796–7813. doi: 10.1007/s12035-024-04019-5, PMID: 38430350

[ref43] Martinowich K. Manji H. Lu B. (2007). New insights into BDNF function in depression and anxiety. Nat. Neurosci. 10, 1089–1093. doi: 10.1038/nn1971, PMID: 17726474

[ref44] Martin-Sanchez D. Fontecha-Barriuso M. Martinez-Moreno J. M. Ramos A. M. Sanchez-Niño M. D. Guerrero-Hue M.. (2020). Ferroptosis and kidney disease. Nefrologia 40, 384–394. doi: 10.1016/j.nefroe.2020.09.006 32624210

[ref45] Mei S. Q. Yu Q. Y. Sun T. Peng R. Long non-coding RNA TUG1 induces ferroptosis in hippocampal neurons and depressive-like behaviors by facilitating the ubiquitination of DUSP14. (2024).

[ref46] Min W. Liu C. Yang Y. Sun X. Zhang B. Xu L.. (2012). Alterations in hypothalamic-pituitary-adrenal/thyroid (HPA/HPT) axes correlated with the clinical manifestations of depression. Prog. Neuro-Psychopharmacol. Biol. Psychiatry 39, 206–211. doi: 10.1016/j.pnpbp.2012.06.017, PMID: 22750689

[ref47] Peng S. Li W. Lv L. Zhang Z. Zhan X. (2018). BDNF as a biomarker in diagnosis and evaluation of treatment for schizophrenia and depression. Discov. Med. 26, 127–136, PMID: 30586536

[ref48] Peng Z. Zhang C. Yan L. Zhang Y. Yang Z. Wang J.. (2020). EPA is more effective than DHA to improve depression-like behavior, glia cell dysfunction and hippcampal apoptosis signaling in a chronic stress-induced rat model of depression. Int. J. Mol. Sci. 21:1769. doi: 10.3390/ijms21051769, PMID: 32150824 PMC7084382

[ref49] Proulx C. D. Hikosaka O. Malinow R. (2014). Reward processing by the lateral habenula in normal and depressive behaviors. Nat. Neurosci. 17, 1146–1152. doi: 10.1038/nn.3779, PMID: 25157511 PMC4305435

[ref50] Qiu Y. Cao Y. Cao W. Jia Y. Lu N. (2020). The application of ferroptosis in diseases. Pharmacol. Res. 159:104919. doi: 10.1016/j.phrs.2020.104919, PMID: 32464324

[ref51] Raison C. L. Capuron L. Miller A. H. (2006). Cytokines sing the blues: inflammation and the pathogenesis of depression. Trends Immunol. 27, 24–31. doi: 10.1016/j.it.2005.11.006, PMID: 16316783 PMC3392963

[ref52] Schildkraut J. J. Draskoczy P. R. Gershon E. S. Reich P. Grab E. L. (1972). Catecholamine metabolism in affective disorders. IV. Preliminary studies of norepinephrine metabolism in depressed patients treated with amitriptyline. J. Psychiatr. Res. 9, 173–185. doi: 10.1016/0022-3956(72)90019-2 5083173

[ref53] Shen J. Hao C. Yuan S. Chen W. Tong T. Chen Y.. (2024). Acupuncture alleviates CUMS-induced depression-like behaviors of rats by regulating oxidative stress, neuroinflammation and ferroptosis. Brain Res. 1826:148715. doi: 10.1016/j.brainres.2023.148715, PMID: 38142722

[ref54] Song Y. Cao H. Zuo C. Gu Z. Huang Y. Miao J.. (2023). Mitochondrial dysfunction: a fatal blow in depression. Biomed. Pharmacother. 167:115652. doi: 10.1016/j.biopha.2023.115652, PMID: 37801903

[ref55] Song X. Long D. (2020). Nrf2 and ferroptosis: a new research direction for neurodegenerative diseases. Front. Neurosci. 14:484266. doi: 10.3389/fnins.2020.00267 PMC7186402 32372896

[ref56] Soytürk H. Bozat B. G. Karakas F. P. Coskun H. Firat T. (2023). Neuroprotective effects of goji berry (*Lycium barbarum* L.) polysaccharides on depression-like behavior in ovariectomized rats: behavioral and biochemical evidence. Croat. Med. J. 64, 231–242. doi: 10.3325/cmj.2023.64.231, PMID: 37654035 PMC10509687

[ref57] Stockwell B. R. (2022). Ferroptosis turns 10: emerging mechanisms, physiological functions, and therapeutic applications. Cell 185, 2401–2421. doi: 10.1016/j.cell.2022.06.003 35803244 PMC9273022

[ref58] Strlic M. Radovič T. Kolar J. Pihlar B. (2002). Anti-and prooxidative properties of gallic acid in Fenton-type systems. J. Agric. Food Chem. 50, 6313–6317. doi: 10.1021/jf025636j, PMID: 12381109

[ref59] Su W. Deng J. Yang L. Wang Y. Gong X. Li J. (2024). Melatonin alleviates LPS-induced depression-like behavior in mice by inhibiting ferroptosis by regulating RNA methylation-mediated SIRT6/Nrf2/HO-1 pathway. Eur. J. Nutr. 63, 3133–3148. doi: 10.1007/s00394-024-03495-8 39294335

[ref60] Sun X. Ou Z. Chen R. Niu X. Chen D. Kang R.. (2016). Activation of the p62-Keap1-NRF2 pathway protects against ferroptosis in hepatocellular carcinoma cells. Hepatology 63, 173–184. doi: 10.1002/hep.28251, PMID: 26403645 PMC4688087

[ref61] Swetlitz N. (2021). Depression’s problem with men. AMA J. Ethics 23, E586–E589. doi: 10.1001/amajethics.2021.586, PMID: 34351273

[ref62] Tang D. Chen X. Kang R. Kroemer G. (2021). Ferroptosis: molecular mechanisms and health implications. Cell Res. 31, 107–125. doi: 10.1038/s41422-020-00441-1, PMID: 33268902 PMC8026611

[ref63] Tang L. J. Zhou Y. J. Xiong X. M. Li N. S. Zhang J. J. Luo X. J.. (2021). Ubiquitin-specific protease 7 promotes ferroptosis via activation of the p53/TfR1 pathway in the rat hearts after ischemia/reperfusion. Free Radic. Biol. Med. 162, 339–352. doi: 10.1016/j.freeradbiomed.2020.10.307, PMID: 33157209

[ref64] Tang Z. J. Zou W. Yuan J. Zhang P. Tian Y. Xiao Z. F.. (2015). Antidepressant-like and anxiolytic-like effects of hydrogen sulfide in streptozotocin-induced diabetic rats through inhibition of hippocampal oxidative stress. Behav. Pharmacol. 26, 427–435. doi: 10.1097/FBP.0000000000000143, PMID: 25932716

[ref65] Tian J. S. Shi B. Y. Xiang H. Gao S. Qin X. M. du G. H. (2013). 1H-NMR-based metabonomic studies on the anti-depressant effect of genipin in the chronic unpredictable mild stress rat model. PLoS One 8:e75721. doi: 10.1371/journal.pone.0075721, PMID: 24058700 PMC3776757

[ref66] Tonon A. C. Pilz L. K. Markus R. P. Hidalgo M. P. Elisabetsky E. (2021). Melatonin and depression: a translational perspective from animal models to clinical studies. Front. Psych. 12:638981. doi: 10.3389/fpsyt.2021.638981, PMID: 33897495 PMC8060443

[ref67] Ursini F. Maiorino M. (2020). Lipid peroxidation and ferroptosis: the role of GSH and GPx4. Free Radic. Biol. Med. 152, 175–185. doi: 10.1016/j.freeradbiomed.2020.02.027, PMID: 32165281

[ref68] Wang X. Li S. Yu J. Wang W. du Z. Gao S.. (2023b). Saikosaponin B2 ameliorates depression-induced microglia activation by inhibiting ferroptosis-mediated neuroinflammation and ER stress. J. Ethnopharmacol. 316:116729. doi: 10.1016/j.jep.2023.116729, PMID: 37277081

[ref69] Wang X. Shao N. Zhang X. Chen H. Chang Z. Xie D.. (2023a). Ferulic acid activates SIRT1-mediated Ferroptosis signaling pathway to improve cognition dysfunction in Wilson’s disease. Neuropsychiatr. Dis. Treat. 19, 2681–2696. doi: 10.2147/NDT.S443278, PMID: 38077239 PMC10710261

[ref70] Wang Y. Wang S. Xin Y. Zhang J. Wang S. Yang Z.. (2021). Hydrogen sulfide alleviates the anxiety-like and depressive-like behaviors of type 1 diabetic mice via inhibiting inflammation and ferroptosis. Life Sci. 278:119551. doi: 10.1016/j.lfs.2021.119551 33945828

[ref71] Wang X. Xiao A. Yang Y. Zhao Y. Wang C. C. Wang Y.. (2022). DHA and EPA prevent seizure and depression-like behavior by inhibiting ferroptosis and neuroinflammation via different mode-of-actions in a pentylenetetrazole-induced kindling model in mice. Mol. Nutr. Food Res. 66:e2200275. doi: 10.1002/mnfr.202200275, PMID: 36099650

[ref72] Wang L. Xu R. Huang C. Yi G. Li Z. Zhang H.. (2023). Targeting the ferroptosis crosstalk: novel alternative strategies for the treatment of major depressive disorder. Gen Psychiatr. 36:e101072. doi: 10.1136/gpsych-2023-101072, PMID: 37901286 PMC10603325

[ref73] Wang Y. Zheng L. Shang W. Yang Z. Li T. Liu F.. (2022). Wnt/beta-catenin signaling confers ferroptosis resistance by targeting GPX4 in gastric cancer. Cell Death Differ. 29, 2190–2202. doi: 10.1038/s41418-022-01008-w, PMID: 35534546 PMC9613693

[ref74] Wenzel S. E. Tyurina Y. Y. Zhao J. St. Croix C. M. Dar H. H. Mao G.. (2017). PEBP1 wardens ferroptosis by enabling lipoxygenase generation of lipid death signals. Cell 171, 628–641.e26. e26. doi: 10.1016/j.cell.2017.09.044, PMID: 29053969 PMC5683852

[ref75] Wu X. Li Y. Zhang S. Zhou X. (2021). Ferroptosis as a novel therapeutic target for cardiovascular disease. Theranostics 11, 3052–3059. doi: 10.7150/thno.54113, PMID: 33537073 PMC7847684

[ref76] Xu S. He Y. Lin L. Chen P. Chen M. Zhang S. (2021). The emerging role of ferroptosis in intestinal disease. Cell Death Dis. 12:289. doi: 10.1038/s41419-021-03559-1, PMID: 33731703 PMC7969743

[ref77] Xu M. Tao J. Yang Y. Tan S. Liu H. Jiang J.. (2020). Ferroptosis involves in intestinal epithelial cell death in ulcerative colitis. Cell Death Dis. 11:86. doi: 10.1038/s41419-020-2299-1, PMID: 32015337 PMC6997394

[ref78] Xu X. Wu Y. Yi K. Hu Y. Ding W. Xing C. (2021). IRF1 regulates the progression of colorectal cancer via interferon-induced proteins. Int. J. Mol. Med. 47, 1–13. doi: 10.3892/ijmm.2021.4937, PMID: 33907823 PMC8054637

[ref79] Xu C. Xiong Q. Tian X. Liu W. Sun B. Ru Q.. (2022). Alcohol exposure induces depressive and anxiety-like behaviors via activating ferroptosis in mice. Int. J. Mol. Sci. 23:13828. doi: 10.3390/ijms232213828, PMID: 36430312 PMC9698590

[ref80] Yagi K. Shidoji Y. Komura S. Kojima H. Ohishi N. (1998). Dissipation of mitochondrial membrane potential by exogenous phospholipid monohydroperoxide and protection against this effect by transfection of cells with phospholipid hydroperoxide glutathione peroxidase gene. Biochem. Biophys. Res. Commun. 245, 528–533. doi: 10.1006/bbrc.1998.8417 9571189

[ref81] Yang Z. Jiang Y. Xiao Y. Qian L. Jiang Y. Hu Y.. (2024). Di-Huang-Yin-Zi regulates P53/SLC7A11 signaling pathway to improve the mechanism of post-stroke depression. J. Ethnopharmacol. 319:117226. doi: 10.1016/j.jep.2023.117226, PMID: 37748635

[ref82] Yang Y. Ma Y. Li Q. Ling Y. Zhou Y. Chu K.. (2022). STAT6 inhibits ferroptosis and alleviates acute lung injury via regulating P53/SLC7A11 pathway. Cell Death Dis. 13:530. doi: 10.1038/s41419-022-04971-x, PMID: 35668064 PMC9169029

[ref83] Yang R. Shi L. Si H. Hu Z. Zou L. Li L.. (2023). Gallic acid improves comorbid chronic pain and depression behaviors by inhibiting P2X7 receptor-mediated ferroptosis in the spinal cord of rats. ACS Chem. Neurosci. 14, 667–676. doi: 10.1021/acschemneuro.2c00532, PMID: 36719132

[ref84] Yang W. S. Stockwell B. R. (2008). Synthetic lethal screening identifies compounds activating iron-dependent, nonapoptotic cell death in oncogenic-RAS-harboring cancer cells. Chem. Biol. 15, 234–245. doi: 10.1016/j.chembiol.2008.02.010, PMID: 18355723 PMC2683762

[ref85] Yao M. Y. Liu T. Zhang L. Wang M. J. Yang Y. Gao J. (2021). Role of ferroptosis in neurological diseases. Neurosci. Lett. 747:135614. doi: 10.1016/j.neulet.2020.135614, PMID: 33485988

[ref86] Yu Z. Chen N. Hu D. Chen W. Yuan Y. Meng S.. (2020). Decreased density of perineuronal net in prelimbic cortex is linked to depressive-like behavior in young-aged rats. Front. Mol. Neurosci. 13:4. doi: 10.3389/fnmol.2020.00004, PMID: 32116542 PMC7025547

[ref87] Zeng T. Li J. Xie L. Dong Z. Chen Q. Huang S.. (2023). Nrf2 regulates iron-dependent hippocampal synapses and functional connectivity damage in depression. J. Neuroinflammation 20:212. doi: 10.1186/s12974-023-02875-x, PMID: 37735410 PMC10512501

[ref88] Zhang G. Lv S. Zhong X. Li X. Yi Y. Lu Y.. (2024). Ferroptosis: a new antidepressant pharmacological mechanism. Front. Pharmacol. 14:1339057. doi: 10.3389/fphar.2023.1339057, PMID: 38259274 PMC10800430

[ref89] Zhang M. Lyu D. Wang F. Shi S. Wang M. Yang W.. (2022). Ketamine may exert rapid antidepressant effects through modulation of neuroplasticity, autophagy, and ferroptosis in the habenular nucleus. Neuroscience 506, 29–37. doi: 10.1016/j.neuroscience.2022.10.015, PMID: 36280022

[ref90] Zhang Y. Zhang J. Feng D. Zhou H. Gui Z. Zheng M.. (2022). IRF1/ZNF350/GPX4-mediated ferroptosis of renal tubular epithelial cells promote chronic renal allograft interstitial fibrosis. Free Radic. Biol. Med. 193, 579–594. doi: 10.1016/j.freeradbiomed.2022.11.002, PMID: 36356714

[ref91] Zhao L. Zhou X. Xie F. Zhang L. Yan H. Huang J.. (2022). Ferroptosis in cancer and cancer immunotherapy. Cancer Commun. 42, 88–116. doi: 10.1002/cac2.12250, PMID: 35133083 PMC8822596

[ref92] Zhou B. Zhu Z. Ransom B. R. Tong X. (2021). Oligodendrocyte lineage cells and depression. Mol. Psychiatry 26, 103–117. doi: 10.1038/s41380-020-00930-0, PMID: 33144710 PMC7815509

[ref93] Zhu Q. Han Y. He Y. Meng P. Fu Y. Yang H.. (2024). Quercetin inhibits neuronal ferroptosis and promotes immune response by targeting lipid metabolism-related gene PTGS2 to alleviate breast cancer-related depression. Phytomedicine 130:155560. doi: 10.1016/j.phymed.2024.155560, PMID: 38815404

[ref94] Zuo C. Cao H. Song Y. Gu Z. Huang Y. Yang Y.. (2022). Nrf2: an all-rounder in depression. Redox Biol. 58:102522. doi: 10.1016/j.redox.2022.102522, PMID: 36335763 PMC9641011

[ref95] Żurawek D. Turecki G. (2021). The miRNome of depression. Int. J. Mol. Sci. 22:11312. doi: 10.3390/ijms222111312, PMID: 34768740 PMC8582693

